# Task-Related Synaptic Changes Localized to Small Neuronal Population in Recurrent Neural Network Cortical Models

**DOI:** 10.3389/fncom.2018.00083

**Published:** 2018-10-05

**Authors:** Satoshi Kuroki, Takuya Isomura

**Affiliations:** ^1^Laboratory for Behavioral Genetics, Center for Brain Science, RIKEN, Wako, Japan; ^2^Laboratory of Network Dynamics of Memory, Center for Functional Connectomics, Korea Institute of Science and Technology, Seoul, South Korea; ^3^Laboratory for Neural Computation and Adaptation, Center for Brain Science, RIKEN, Wako, Japan

**Keywords:** recurrent neural network, plasticity, synaptic weight, sparseness, cognitive flexibility, prefrontal cortex

## Abstract

Humans have flexible control over cognitive functions depending on the context. Several studies suggest that the prefrontal cortex (PFC) controls this cognitive flexibility, but the detailed underlying mechanisms remain unclear. Recent developments in machine learning techniques allow simple PFC models written as a recurrent neural network to perform various behavioral tasks like humans and animals. Computational modeling allows the estimation of neuronal parameters that are crucial for performing the tasks, which cannot be observed by biologic experiments. To identify salient neural-network features for flexible cognition tasks, we compared four PFC models using a context-dependent integration task. After training the neural networks with the task, we observed highly plastic synapses localized to a small neuronal population in all models. In three of the models, the neuronal units containing these highly plastic synapses contributed most to the performance. No common tendencies were observed in the distribution of synaptic strengths among the four models. These results suggest that task-dependent plastic synaptic changes are more important for accomplishing flexible cognitive tasks than the structures of the constructed synaptic networks.

## Introduction

Human brains can quickly generate, and flexibly switch between, sensory-sensory and sensory-motor associations depending on the situation, even in the same environment. The prefrontal cortex (PFC) controls cognitive flexibility (also known as executive function) (Miller and Cohen, 2001; Nobre and Kastner, 2014). Although numerous studies have examined the control mechanisms of executive function using animal models, the details remain unclear. One reason for this is the limited number of biologic variables of the brain that can be observed and manipulated.

By contrast, computational modeling allows investigators to track detailed transitions of variables during a task. Recent developments in machine learning have established learning rules for simple recurrent neural networks (RNNs) to perform various tasks (Jaeger and Haas, 2004; Sussillo and Abbott, 2009; Laje and Buonomano, 2013). In fact, the activities of the PFC while performing flexible cognitive tasks can be modeled using RNNs (Mante et al., 2013; Song et al., 2016, 2017; Miconi, 2017).

**Figure 1:**
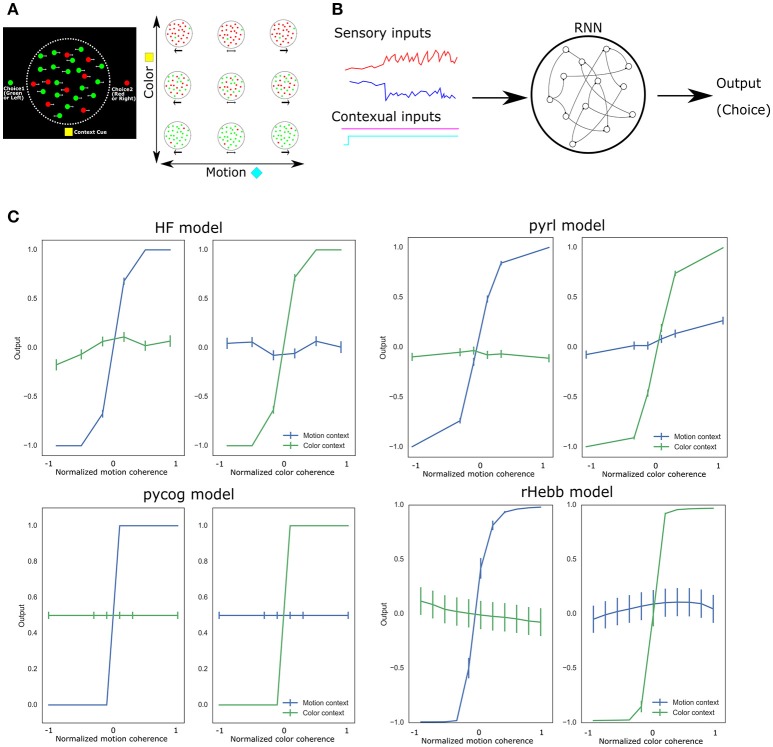
Experimental design and task performance of each model. **(A)** Context-dependent integration task applied to a monkey. The left panel shows a schematic representation of the task. At the center of the screen, the green or red dots move randomly from right to left. The monkey should choose between the left (green) or right (red) option by saccade while referring to the central moving dots. The reference (color or motion) for the correct answer depended on the contextual cue at the bottom of the screen. The right panel indicates a variety of colored moving dot patterns and contextual cues. **(B)** Schematic image of RNN PFC models. All models comprise sensory and contextual inputs and output choices. The input and output representations were simplified in a few dimensions. In the RNN, the neuronal units are connected to each other. **(C)** Task performance of each system after learning. Vertical bars represent standard error of the mean (SEM).

Mante et al. (2013) compared the neural population activity of the PFC in monkey and RNN models during a flexible cognitive task. To evaluate cognitive flexibility, they modified a random-dot motion task by increasing the salience of color (Mante et al., 2013), which is referred to as the context-dependent integration task (Song et al., 2016). To perform this task, the subject monkey must select one of two options based on colored dots moving randomly across a screen. In the task, depending on the contextual cues, the monkey selects the appropriate answer based on either the color or the motion (Figure 1A). The contextual cues randomly change from trial to trial. Mante et al. (2013) then constructed an RNN model that could perform the task, wherein the population activities during the task were similar to those of the monkey's PFC neurons. In the RNN model, however, they optimized the synaptic strengths using the Hessian-free (HF) approach, referred to as the “HF model” (Martens, 2010; Martens and Sutskever, 2011), which is not sufficiently biologically plausible.

Aiming for more biologically reasonable models, several groups have suggested RNN models for context-dependent integration tasks. Song et al. proposed an RNN model termed the “pycog model” (Song et al., 2016), which consists of separate excitatory and inhibitory neuronal units and employs a simpler learning rule than the HF model. Briefly, the basic principle is based on a modified stochastic gradient descent (SGD) method (Pascanu et al., 2013). In addition to context-dependent integration tasks, the pycog model allows for the investigation of several PFC-dependent behavioral tasks.

Moreover, Song et al. (2017) developed another RNN model, referred to as the “pyrl model,” which comprises a policy network that selects the next behaviors and a baseline network that evaluates future rewards, by which learning is reinforced with reward signals (Song et al., 2017). The pyrl model is the so-called actor-critic method (Sutton and Barto, 1998) built with a policy gradient reinforcement learning rule known as the REINFORCE algorithm (Williams, 1992; Wierstra et al., 2010). The baseline network optimizes the output to predict future rewards in each context, whereas the policy network learns to make an optimal choice to maximize future rewards.

In addition to the above-described RNN models, Miconi introduced the reward-modulated Hebbian rule abbreviated as the “rHebb model” (Miconi, 2017). This model utilizes the node-perturbation method (Fiete et al., 2007) and is biologically more plausible than the HF or SGD models. This system also performs several cognitive tasks.

In the present study, we compared the synaptic weight structures of the four RNN models (HF, pycog, pyrl, and rHebb) while performing context-dependent integration tasks (Figure 1B). Interestingly, in the plastic changes of the synaptic weights from the initial network state to the last learned state, all models showed that the plastic synapses were localized to small populations of neuronal units and the projections to a few postsynaptic neurons were highly plastic. The highly plastic units made greater contributions to performed behaviors than the low plastic units in the HF, pycog, and pyrl models, but not in the rHebb model. In addition, the distributions of the synaptic weight changes exhibited a large positive kurtosis in most of the models. No tendencies in the synaptic strengths of the networks were observed after learning the task (i.e., constructed networks). The present results indicate that plastic changes induced by task learning are more important than the constructed network structures of the system.

## Materials and methods

### Model descriptions

The parameter settings were set to default values based on previous reports and scripts (Mante et al., 2013; Song et al., 2016, 2017; Miconi, 2017). The HF, pycog, and rHebb models were expressed by the following equation:

(1) $$\begin{array}{l} \tau \overset{\cdot }{x}\left(t\right)=-x\left(t-1\right)+W_{rec}r\left(t-1\right)+W_{in}u\left(t\right)+b_{x}+\rho_{x}\left(t\right) \end{array}$$

where, τ > 0 is the time constant, $x\left(t\right)\in {\mathbb{R}}^{N_{rec}}$ corresponds to the membrane potentials of recurrent neuronal units at discrete time step *t*, $r\left(t\right)\in {\mathbb{R}}^{N_{rec}}$represents the firing rate calculated by the rectified linear activation function *r*(*t*) = *x*(*t*) *for x*(*t*) > 0 *and r*(*t*) = 0 *otherwise* for the pycog and pyrl models; or the hyperbolic tangent function *r*(*t*) = tanh (*x*(*t*)) for the HF and rHebb models, where *N*_*rec*_ is the number of recurrent units. Moreover, $u\left(t\right)\in {\mathbb{R}}^{N_{in}}$ is an (external) task input comprising sensory and contextual information; $W_{rec}\in {\mathbb{R}}^{N_{rec}\times N_{rec}}$ and $W_{in}\in {\mathbb{R}}^{N_{rec}\times N_{in}}$ are the synaptic weight matrices from recurrent and task inputs to each recurrent unit, respectively; $b_{x}\in {\mathbb{R}}^{N_{rec}}$ is the offset constant of recurrent units; and $\rho_{x}\left(t\right)\in {\mathbb{R}}^{N_{rec}}$ is the noisy fluctuation of each unit following a Gaussian distribution, where *N*_*in*_ corresponds to the number of input units (four channels in the HF and rHebb models and six channels in the pycog and pyrl models). For the pyrl model, we used a gated recurrent unit (Chung et al., 2014) for which *Equation 1* was modified (see section pyrl Model). Note that we used *N*_*rec*_ = 100 for HF, *N*_*rec*_ = 150for pycog (120 excitatory plus 30 inhibitory units), and *N*_*rec*_ = 200 for rHebb. For pyrl, we used *N*_*rec*_ = 100 for the policy network and *N*_*rec*_ = 100 for the baseline network.

The readout units of HF, pycog, and pyrl were given by:

(2) $$\begin{array}{l} z\left(t\right)=W_{out}r\left(t\right)+b_{z} \end{array}$$

where, $z\left(t\right)\in {\mathbb{R}}^{N_{out}}\;$ is the output of the system, *N*_*out*_ is the number of output units (one channel in the HF model, two channels in the pycog model, and three channels in the pyrl model), $W_{out}\in {\mathbb{R}}^{N_{out}\times N_{rec}}$is the synaptic weight matrix from recurrent units to readout units, and $b_{z}\in {\mathbb{R}}^{N_{out}}$ is the offset constant of the readout unit. In contrast, the rHebb model used an arbitrary recurrent unit as an output. The choices of the system were represented as the signs of the output unit [*z*(*t*) in the HF model and the arbitrary unit *r*(*t*) in the rHebb model; one channel in total] or the highest channel (among two channels in the pycog model and three channels in the pyrl model) of the output units. Only the pyrl model had another choice (like “stay”) in addition to choice 1 and choice 2. The *N*_*in*_, *N*_*rec*_, and *N*_*out*_ of each model are summarized in Table 1.

**Table 1:** Number of input, recurrent, and output units in each model.

**Model**	***N*_*in*_**	***N*_*rec*_**	***N*_*out*_**
HF	4	100	1
pycog	6	150 (Ex: 120, Inh: 30)	2
pyrl (policy)	6	100	3
rHebb	4	200	1 (an arbitrary unit)

### Task descriptions

The task inputs, *u*(*t*)in *Equation 1*, comprise two sets of sensory and two sets of contextual information. Sensory inputs were defined as:

(3) $$\begin{array}{lll} u_{m}\left(t\right) & = & d_{m}+\rho_{m}\left(t\right) \end{array}$$

(4) $$\begin{array}{lll} u_{c}\left(t\right) & = & d_{c}+\rho_{c}\left(t\right) \end{array}$$

where $u_{m}\left(t\right)\in {\mathbb{R}}^{1\;or\;2}$ and $u_{c}\left(t\right)\in {\mathbb{R}}^{1\;or\;2}$ are the motion and color sensory inputs, respectively, $d_{m}\in {\mathbb{R}}^{1\;or\;2}$ and $d_{c}\in {\mathbb{R}}^{1\;or\;2}$ are the offsets, and $\rho_{m}\left(t\right)\in {\mathbb{R}}^{1\;or\;2}$ and $\rho_{c}\left(t\right)\in {\mathbb{R}}^{1\;or\;2}$ are Gaussian noises with a zero mean. The amplitudes *d*_*m*_ and *d*_*c*_ represent motion and color coherence. Input features (e.g., right and left in motion, red and green in color; see Figure 1A) were represented by a plus or minus sign for *d*_*m*_ (e.g., right +, left–) and *d*_*c*_ (e.g., red +, green –) in the HF and rHebb models (two channels in total). These input features were represented as independent channel inputs in the pycog and pyrl models (four channels in total). In addition, the contextual information was modeled with a set of two binary inputs, *u*_*cm*_(*t*) ∈ {0, 1} and *u*_*cc*_(*t*) ∈ {0, 1}, where *u*_*cm*_(*t*) = 1 and *u*_*cc*_(*t*) = 0 in the motion context and *u*_*cm*_(*t*) = 0 and *u*_*cc*_(*t*) = 1 in the color context at every time-step*t*.

### HF model

The HF model was implemented based on a previous study (Mante et al., 2013). HF optimization was mounted with modifying scripts written by Boulanger-Lewandowski and available on Github (https://github.com/boulanni/theano-hf) (Boulanger-Lewandowski et al., 2012).

HF optimization (Shewchuk, 1994; Martens, 2010; Martens and Sutskever, 2011) was processed by minimizing the following objective function E(θ),

(5) $$\begin{array}{l} \varepsilon \left(\theta \right)=L\left(\theta \right)+\lambda_{R}R\left(\theta \right) \end{array}$$

where each component is given by:

(6) $$\begin{array}{l} L\left(\theta \right)=\frac{1}{2N_{trial}}\overset{N_{trial}}{\underset{n=1}{\sum }}\underset{t=0,T}{\sum }{\left(z_{n}\left(t,\theta \right)-z_{n}^{target}\left(t\right)\right)}^{2} \end{array}$$

(7) $$\begin{array}{l} R\left(\theta \right)\;=\;D\left(r,\theta \right),r\left(\theta \;+\;\Delta \;\theta \right) \end{array}$$

Note that θ is the vectorized parameter (a set of *W*_*rec*_, *W*_*in*_, *W*_*out*_, *b*_*x*_, *and b*_*z*_). Function *L*(θ) indicates the error between the target $z_{n}^{target}\left(t\right)$ and the actual output *z*_*n*_(*t*, θ) of the system at the first and last time-points (*t* = 0, *T*) of all the trials (*N*_*trial*_). The target outputs $z_{n}^{target}\left(t\right)$ of the last time-points (*t* = *T*) presented a choice by setting 1 or −1, which corresponded with choice 1 and choice 2, respectively. The target outputs $z_{n}^{target}\left(t\right)$ were initialized to 0. Function *R*(θ) indicates structural damping to prevent disruptive changes of recurrent unit activities, because even a small perturbation of the recurrent units can result in a very large output difference (Martens and Sutskever, 2011). *D*(*r*(θ), *r*(θ + Δθ)) is the distance (cross entropy in our script) between outputs of recurrent units with parameter θ and θ + Δθ. Coefficient λ_*R*_ > 0 determines the degree of the *R*(θ) penalty, and its value is determined using the Levenberg-Marquardt algorithm (Nocedal and Wright, 1999). An optimal θ that gives a minimum object function was resolved with HF optimization (Martens, 2010; Martens and Sutskever, 2011).

The dimensions of *N*_*in*_ = 4, *N*_*rec*_ = 100, and *N*_*out*_ = 1 were used in the HF model. Because of the memory capacity, the duration of the task was 25 times shorter (30 ms) than the original setting (750 ms). The standard deviations of noises were reduced from the original values in Mante et al. (2013) and defined such that *Std*[ρ_*x*_] = 0.004 in *Equation 1*, *Std*[ρ_*m*_] = 0.04 in *Equation 3*, and *Std*[ρ_*c*_] = 0.04 in *Equation 4*. The time constant τ = 10 ms and the time-step Δt = 1 ms. The initial weight distribution of the default setting was a Gaussian distribution (mean = 0, standard deviation = 0.01) and later tested with a different setting (Gaussian distribution, mean = 0, standard deviation = 0.15) to make the initial weight setting comparable to that in the other models in Supplementary Figure 4.

### Pycog model

The pycog model was obtained from Github (https://github.com/xjwanglab/pycog) and run in its original setting as described previously (Song et al., 2016). This system learns tasks with a modified SGD (Pascanu et al., 2013) by minimizing the following objective function E(θ),

(8) $$\begin{array}{l} E\left(\theta \right)=\frac{1}{N_{trials}}\overset{N_{trials}}{\underset{n=1}{\sum }}\left(L_{n}\left(\theta \right)+\lambda_{\Omega }\Omega_{n}\left(\theta \right)\right) \end{array}$$

where θ is the vectorized parameter set for the optimization, *N*_*trials*_is the number of trials, and *L*_*n*_(θ) is the error between the actual and target outputs ($z_{s}\left(t,\theta \right)\;\in {\mathbb{R}}^{2}$ and $z_{s}^{target}\left(t,\theta \right)\in {\mathbb{R}}^{2}$, respectively) through trial (*T*) and the number of output units (*N*_*out*_) given by

(9) $$\begin{array}{l} L_{n}\left(\theta \right)=\frac{1}{N_{out}T}\overset{N_{out}}{\underset{s=1}{\sum }}\overset{T}{\underset{t=1}{\sum }}M_{t}^{error}{\left[z_{s}\left(t,\theta \right)-z_{s}^{target}\left(t\right)\right]}^{2} \end{array}$$

where $M_{t}^{error}\in \left\{0,1\right\}$ is the error mask consisting of 0 or 1 and determines whether the error at time-point *t* should be taken into account (in a context-dependent integration task; only the last output is considered). Ω_*n*_(θ) in *Equation 8* is a regularization term used to preserve the size of the gradients as errors are propagated through time, and λ_Ω_ determines the effects of the regularization.

The values *N*_*in*_ = 6, *N*_*rec*_ = 150, and *N*_*out*_ = 2 were used in the pycog model. Of note, this system includes both excitatory and inhibitory units at an excitatory to inhibitory ratio of 4:1, indicating that the number of excitatory units is 120 and that of inhibitory units is 30. For our network analyses, we mainly used excitatory-excitatory (E-E) connections. The initial weight distribution of the default setting is a gamma distribution and the multiplier is positive or negative depending on the input unit type (excitatory or inhibitory). Applying a uniform distribution as an initial weight distribution, the minimum and maximum are 0 and 1, respectively.

### pyrl model

The pyrl model was also obtained from Github (https://github.com/xjwanglab/pyrl) and run in its original setting (Song et al., 2017). The network consists of policy and baseline RNN, in which the nodes are gated recurrent units (Chung et al., 2014). The policy network aims to maximize the expected future rewards, which are optimized using the REINFORCE algorithm (Williams, 1992; Wierstra et al., 2010).

(10) $$\begin{array}{l} E^{\pi }\left(\theta \right)=\frac{1}{N_{trials}}\overset{N_{trials}}{\underset{n=1}{\sum }}\left[-J_{n}\left(\theta \right)+\Omega_{n}^{\pi }\left(\theta \right)\right] \end{array}$$

(11) $$\begin{array}{l} J_{n}\left(\theta \right)=E_{H}\left[\overset{T}{\underset{t=0}{\sum }}R_{t+1}\right] \end{array}$$

where θ is the vectorized parameter set of the policy network for the optimization, $E^{\pi }\left(\theta \right)$is the objective function of the policy network, *N*_*trials*_ is the number of trials, *J*_*n*_(θ)is the expected reward prediction, $\Omega_{n}^{\pi }\left(\theta \right)$ is regularization term used to preserve the size of the gradients as errors are propagated through time (Song et al., 2016), and *E*_*H*_ represents the expectation of reward *R*_*t*_ over all possible trial histories *H*.

The baseline network minimizes the difference between the actual and estimated reward values throughout the trial.

(12) $$\begin{array}{l} E^{v}\left(\phi \right)=\frac{1}{N_{trials}}\overset{N_{trials}}{\underset{n=1}{\sum }}\left[E_{n}\left(\phi \right)+\Omega_{n}^{v}\left(\phi \right)\right] \end{array}$$

(13) $$\begin{array}{l} E_{n}\left(\phi \right)=\frac{1}{T+1}\overset{T}{\underset{t=0}{\sum }}{\left[\overset{T}{\underset{\tau =t}{\sum }}R_{\tau +1}-v_{\phi }\left(\pi_{1:t},r_{1:t}^{\pi }\right)\right]}^{2} \end{array}$$

where ϕ is the vectorized parameter set of the baseline network for the optimization, $E^{v}\left(\phi \right)$ is the objective function of the baseline network, *E*_*n*_(ϕ) is the error between the actual reward *R*_τ_ (a correct decision is rewarded with *R*_τ_ = 1, if incorrect *R*_τ_ = 0, and the duration of breaking the fixation before the decision is negatively rewarded with *R*_τ_ = −1), $v_{\phi }\left(\pi_{1:t},r_{1:t}^{\pi }\right)$ is the expected (readout) reward prediction of the baseline network under recurrent unit activities ($r_{1:t}^{\pi }$) and choice (π_1:*t*_) of the policy network through a trial (*T*), and $\Omega_{n}^{v}\left(\theta \right)$ is a regularization term of the baseline network. This system is optimized using Adam SGD (Kingma and Ba, 2015) with gradient clipping (Pascanu et al., 2013).

The values *N*_*in*_ = 6 (task inputs), *N*_*rec*_ = 100, and *N*_*out*_ = 3 (choice) were used in the policy network, and the values *N*_*in*_ = 103 [*r*(*t*)and π(*t*) of the policy network], *N*_*rec*_ = 100, and *N*_*out*_ = 1 (readout reward prediction) were used in the baseline network. We used the policy network for the main analysis of this study because the baseline network was not involved in performing the task (the baseline network is critical for optimizing the system). The default initial weight distribution was obtained from the gamma distribution (*K* = 4) with random multipliers in both policy and baseline networks. The plastic synapses were set at 10% of all synapses and the other synaptic weights were fixed as the defaults throughout the training. We only used plastic synapses for the weight-change distribution analysis. This system had three output choices (choice 1, choice 2, or stay) although the other models had only two choices (choice 1 or choice 2). When a normal distribution was used as an initial weight distribution in Supplementary Figure 4, the mean and standard deviation were 0 and 0.15, respectively.

### rHebb model

The rHebb model was obtained from GitHub (https://github.com/ThomasMiconi/BiologicallyPlausibleLearningRNN) and basically run in its original setting (Miconi, 2017). The network pools Hebbian-like activity in every time-step as follows:

(14) $$\begin{array}{l} e_{i,j}\left(t\right)=e_{i,j}\left(t-1\right)+S\left(r_{j}\left(t-1\right)*\left(x_{i}\left(t\right)-\bar{x_{i}}\right)\right) \end{array}$$

where *e*_*i, j*_(*t*) is the accumulated eligibility trace of the synapse *i* (pre) and *j* (post), *S* is the monotonic superlinear function (in this case *S* = *x*^3^), *r*_*j*_(*t*) is the output of unit *j*, *x*_*i*_(*t*) is the membrane potential of unit *i* at time *t*, and $\bar{x_{i}}$ is the short-time running average of *x*_*i*_. The synaptic weights are modulated with the pooled value and reward error at the end of every trial as follows:

(15) $$\Delta W_{i,j}=\eta e_{i,j}(R-\bar{R})$$

where Δ*W*_*i, j*_ is the change in synaptic weight between *i* and *j*, η is the learning rate, *R* is the reward from the environment (absolute difference from an optimal response is supplied as a negative reward), and $\bar{R}$ is the average of the previously received reward. Three units are used as constant input units in default. The output and constant input units are excluded from the weight-change distribution analysis.

The values *N*_*in*_ = 4 and *N*_*rec*_ = 200 and *N*_*out*_ = 1 were used in the rHebb model. The output of this system is an activity of an arbitrarily chosen unit from the recurrent units. The initial weight distribution of the default setting is a Gaussian distribution (mean = 0, standard deviation = 0.106) and later tested by setting *N*_*rec*_ and the standard deviation of the initial weight Gaussian distribution to 100 and 0.15, respectively, to make the initial weight setting comparable to that in the other models in Supplementary Figure 4.

### Post-mean weights and weight changes

**Figure 2:**
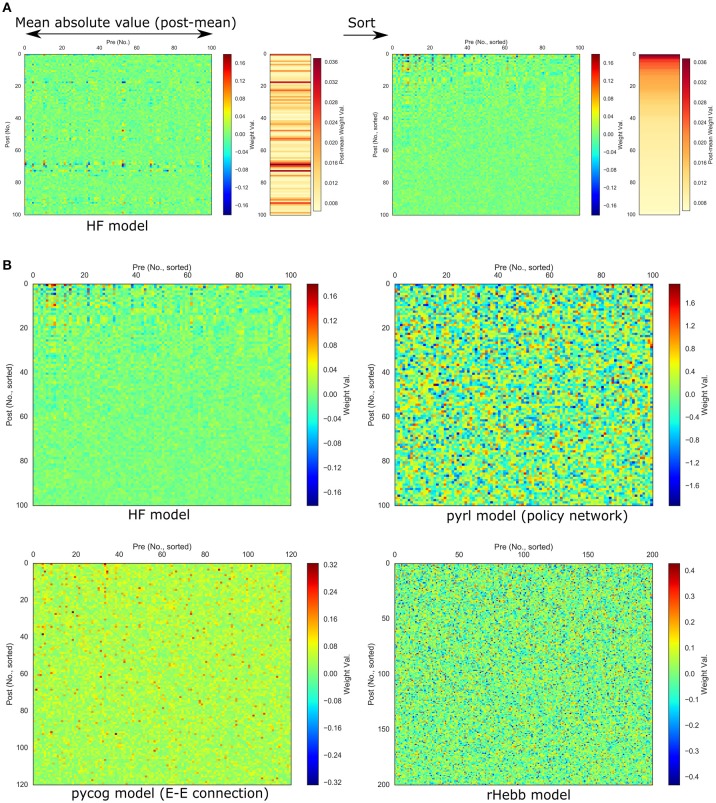
Synaptic weights after task learning. **(A)** Calculation and sorting of post-mean weight value. The extreme left panel shows the synaptic weight values from pre- (horizontal axis) to post-unit (vertical axis) in the HF model after task learning. Post-mean weights were calculated with the mean absolute weight values along the pre-axis (bidirectional-headed arrow). The neighboring right-hand panel shows the post-mean weights of the left panel. Neuronal units sorted by the post-mean values. **(B)** Weight values of each model sorted by post-mean weights.

To quantify the concentration degree of the synaptic weights or the weight changes in each neuronal unit, we calculated the averages of the absolute values that project to each neuron, referred to as the post-mean weights or post-mean weight changes, respectively. Post-mean weights are defined (Figure 2A) by,

(16) $$\begin{array}{l} {\bar{W}}_{i}^{last}=\frac{1}{N}\overset{N}{\underset{j=1}{\sum }}|W_{i,j}^{last}|\;, \end{array}$$

where, $|W_{i,j}^{last}|\;$ is an absolute synaptic weight of the learned model, *j* is an index of a presynaptic recurrent unit projecting to postsynaptic recurrent unit *i*, and *N* is the number of recurrent units of the model. The post-mean weight changes are defined by

(17) $$\begin{array}{l} {\bar{W}}_{i}^{diff}=\frac{1}{N}\overset{N}{\underset{j=1}{\sum }}|W_{i,j}^{diff}|, \end{array}$$

(18) $$\begin{array}{l} W_{i,j}^{diff}=W_{i,j}^{last}-W_{i,j}^{init}, \end{array}$$

where $W_{i,j}^{last}$ and $W_{i,j}^{init}$ are the learned last and initial synaptic weights of the model, respectively.

### Neuronal unit inactivation

The scripts were modified as shown below. Selected unit outputs were set to 0 (pycog, pyrl, and rHebb model) or a constant value (HF model,*b*_*x*_) in every recurrent loop. The number of inactivation units was increased in increments of 10 and sorted in ascending, descending, or shuffled order of the post-mean synaptic weight changes (Figure **6A**). The trial settings, including the number of trials and offset and noise settings of sensory inputs, also followed default conditions in each script.

### Task learning with smaller network

Models learned the context-dependent integration task with modification of the number of recurrent neuronal units *N*_*rec*_. The learning was repeated with five different random seeds in each condition. The Kruskal–Wallis test and Dunn's test were applied for statistics and *post-hoc* multiple comparisons, respectively. The trial settings, including the number of trials and offset and noise settings of sensory inputs, also followed the default conditions in each script.

### Statistical analysis of distributions

Python libraries, numpy, scipy, statsmodels, matplotlib, seaborn, and Jupyter were used for statistical analysis. The Shapiro-Wilk normality test, implemented as a scipy function, was applied to evaluate the normality of the distributions. Kurtosis and skewness were tested using the scipy functions, stats.kurtosistest and stats.skewtest (https://docs.scipy.org). One-way analysis of variance (ANOVA), two-way ANOVA, and multiple comparisons (Tukey honestly significant difference) were performed with the python library, statsmodels (http://www.statsmodels.org). The Kruskal-Wallis test and multiple comparison test (Dunn's test) were performed with functions in the scipy and scikit-posthoc (https://pypi.python.org/pypi/scikit-posthocs) libraries, respectively.

## Results

### Analysis of connection strengths after the learning task

We first confirmed that all four RNN systems successfully learned the context-dependent integration task (Figure 1C). They showed psychometric curves (relationship between the sensory inputs and behavioral responses), which changed depending on the context of the inputs. More than 85% of the choices of all models were correct.

Next, the synaptic weight values of each learned system were analyzed. We used E-E connections in the pycog model for the analysis because this is the dominant connection in the model. Additionally, we used the policy network in the pyrl model because the baseline network was not related to the choice behavior even though it is important for learning the task (see Materials and Methods section). For the HF and rHebb models, we used all synaptic connections between recurrent neuronal units for the analysis. We detected a pattern in the HF model indicating that high negative or positive weights were concentrated in a small number of neuronal units, particularly postsynaptically. To evaluate the high weight concentration in all four models, we calculated means of absolute weight values for each post-unit (post-mean weight, see Materials and Methods section) and sorted postsynaptic neurons in descending order of the post-mean weight (Figure 2A). Figure 2B illustrates synaptic weight distributions of the four models. We confirmed that high post-mean weights localized to a few neuronal units in the HF model, but the postsynaptic neurons in the other three models did not exhibit this localization.

**Figure 3:**
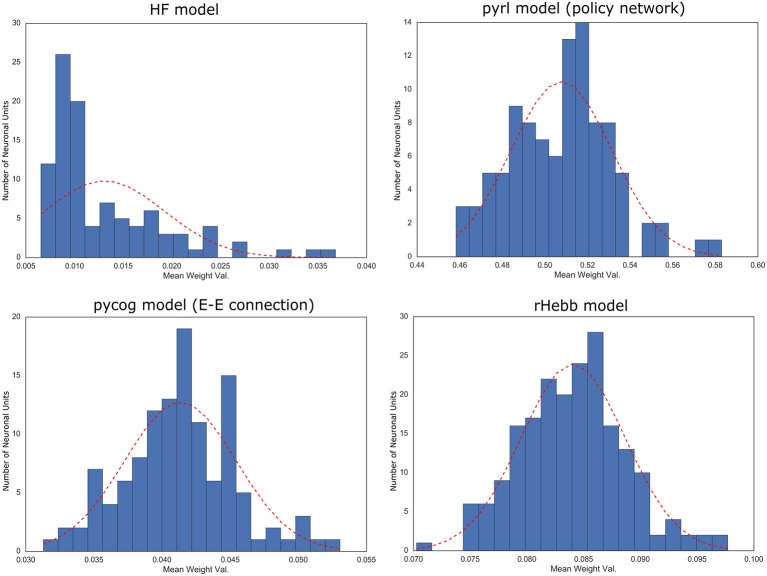
Distribution of post-mean weights after the task learning. Dotted red line indicates normal distribution with mean and standard deviation of the values as reference.

To quantify the weight localization in a sparse population, we analyzed the distribution of the post-mean weight values. The distribution of the HF model was highly skewed (Figure 3, Table 2), indicating that the positive or negative high weights were significantly concentrated in a small number of units. In contrast, the other three models did not show significant skewness. These results suggest that the distribution of the constructed network was not extremely important for performing the task.

**Table 2:** Distribution properties in post-mean weights after task learning.

**Model**	**n**	**Normality**		**Skewness**			**Kurtosis**		
		**P**	**W**	**p**	**Z**	**Skew**.	**p**	**Z**	**kurt**.
HF	100	0.00	0.81	0.00	5.33	1.64	0.00	3.21	2.53
pycog (E-E)	120	0.60	0.99	0.39	0.85	0.81	0.48	0.71	0.19
pyrl (policy)	100	0.23	0.98	0.26	1.13	0.26	0.34	0.95	0.32
rHebb	196	0.55	0.99	0.34	0.96	0.16	0.34	0.95	0.26

Moreover, we investigated the distribution of the synaptic weights over all neurons (Supplementary Figure 1, Supplementary Table 1). Synaptic weights in both the HF and rHebb models were initialized to follow a Gaussian distribution. The HF system showed a non-Gaussian high-kurtosis distribution after task learning, whereas rHebb still showed a Gaussian distribution. The synaptic weight distributions in the pycog and pyrl models did not show Gaussian distributions even from the initial states (see the Materials and Methods section). Thus, no similarities were observed among the models in the weight distribution parameter. Overall, we observed no common tendencies in the constructed network structures across all four models, although all of them succeeded at learning the task.

### Analysis of plastic changes with task learning

**Figure 4:**
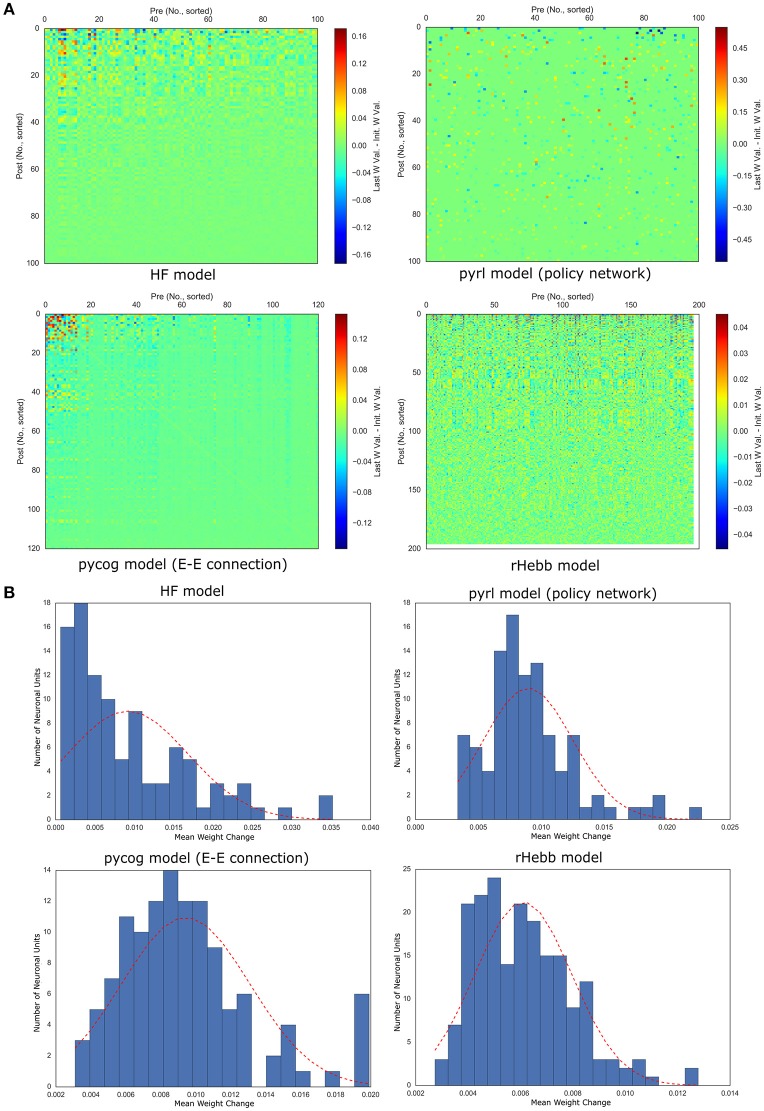
Weight changes induced by task learning. **(A)** Color plots of weight changes sorted by post-mean weight changes. **(B)** Distribution of post-mean weight changes. The dotted red line indicates a normal distribution of the mean and standard deviation of the values as reference.

We then analyzed synaptic weight changes by task learning and their post-mean. The synaptic weight changes were defined as the difference between the initial and final weight values (see Materials and Methods section). Only 10% of the synapses in the pyrl model were analyzed because only those synapses were variable while the others were fixed through learning (as a default setting). We found that in all models, the weight changes were localized to a few units (Figure 4A). Quantitative analyses of the distribution of the post-mean weight changes revealed that all models exhibited highly positively skewed distributions (Figure 4B, Table 3). The pycog model also displayed an inhibitory network, whereas the pyrl model displayed a baseline network. Most of these connections tended to show that the synaptic changes were localized to restricted populations of neuronal units (Supplementary Figure 2). These results indicate that a small number of high-plasticity units largely contributed to the learning in all four networks.

**Table 3:** Distribution properties in post-mean weight changes with task learning.

**Model**	**n**	**Normality**		**Skewness**			**Kurtosis**		
		**p**	**W**	**p**	**Z**	**skew**.	**p**	**Z**	**kurt**.
HF	100	0.00	0.87	0.00	4.44	1.25	0.04	2.08	1.16
pycog (E-E)	120	0.00	0.93	0.00	4.09	1.01	0.05	1.97	0.97
pyrl (policy)	100	0.00	0.91	0.00	4.54	1.29	0.00	3.06	2.31
rHebb	196	0.00	0.96	0.00	4.16	0.78	0.07	1.81	0.68

**Figure 5:**
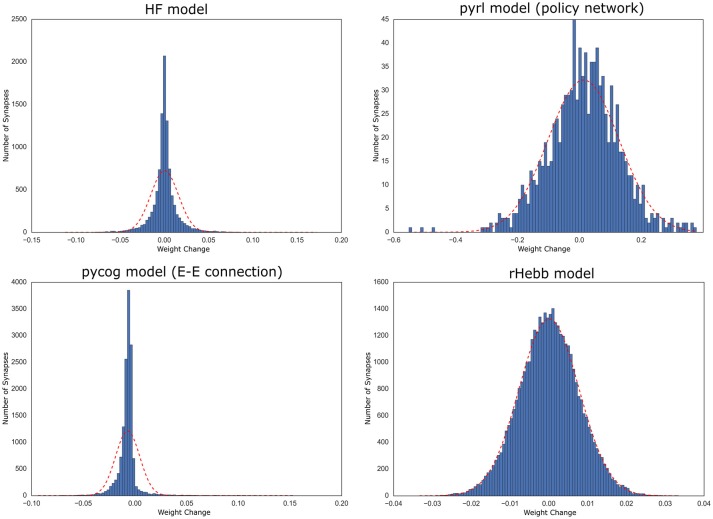
Distribution of weight changes with task learning. The dotted red line indicates a normal distribution of the mean and standard deviation of the values.

We also analyzed the distribution of the weight change over all neurons and observed that these distributions of all four models tended to exhibit positive kurtosis. While the shapes of the distributions of the pyrl and rHebb models were close to a Gaussian distribution, they still had significantly positive kurtosis (Figure 5, Table 4).

**Table 4:** Distribution properties of weight changes with task learning.

**Model**	**n**	**Normality**		**Skewness**			**Kurtosis**		
		**p**	**W**	**p**	**Z**	**skew**.	**p**	**Z**	**kurt**.
HF	10,000	0.00	0.83	0.00	22.6	0.60	0.00	43.1	11.1
pycog (E-E)	14,400	0.00	0.62	0.00	85.4	3.40	0.00	66.1	36.8
pyrl (policy)	1,000	0.00	0.99	0.00	−2.82	−0.22	0.00	5.30	1.24
rHebb	38,416	0.00	0.99	1.00	0.01	0.00	0.00	6.04	0.16

Therefore, all models commonly exhibited high positive skewness in the post-mean weight-change distributions and high kurtosis in the weight-change distributions. These results indicate that plastic changes in all models had long-tailed distributions at both the synapse and neuronal unit levels.

**Figure 6:**
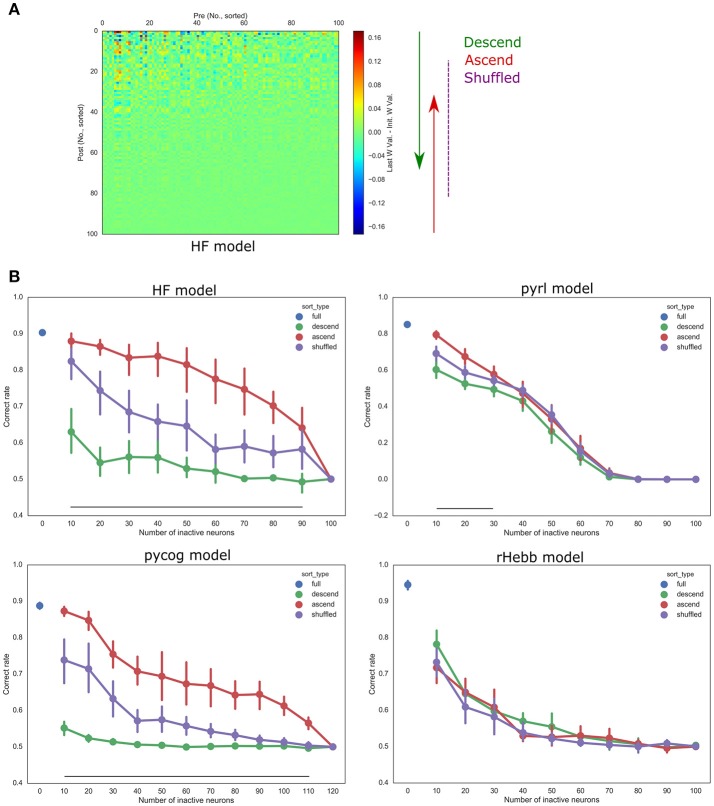
Inactivation experiments. **(A)** The orders of inactivation neuronal units. Left panel shows weight changes in the HF model from Figure 5A. We inactivated units with descending (from high plastic units, green), ascending (from low plastic units, red), or shuffled order (purple). **(B)** Accuracies of task performances of each model. The vertical bars represent the SEM. Black line indicates a significant difference between the descending and ascending order conditions corresponding to the number of inactive neurons.

Furthermore, we validated whether units with higher plasticity had higher contributions to behavior performances. The fixed number of units in each model was inactivated (n_inact) while performing the behavioral task with ascending (starting from low-plasticity units), descending (starting from high-plasticity units), and shuffled order (sort_type) based on the post-mean weight-change values (Figure 6, Table 5). The “n” in Table 5 indicates the number of systems used for the test with different initial settings from different random seeds. All the models showed significant differences in behavior performances among sort_type x n_inact interaction and/or sort_type in a two-way ANOVA, but there were no significant differences among sort types in the rHebb model with *post-hoc* multiple comparisons. These findings indicate that units with higher plasticity in the HF, pycog, and pyrl models made larger contributions to task performance; however, the rHebb model presented redundancy for the loss of the high-plasticity units.

**Table 5:** Two-way ANOVA results of the inactivation experiments.

**Model**	**Test**	***df* (factor)**	***df* (error)**	***F***	***p***
HF (*n* = 11)	n_inact	9	300	34.9	0.00
	sort_type	2	300	231	0.00
	n_inact × sort_type	18	300	4.87	0.00
pycog (*n* = 11)	n_inact	11	360	50.6	0.00
	sort_type	2	360	355	0.00
	n_inact × sort_type	22	360	9.63	0.00
pyrl (*n* = 20)	n_inact	9	570	596	0.00
	sort_type	2	570	24.6	0.00
	n_inact × sort_type	18	570	3.16	0.00
rHebb (*n* = 20)	n_inact	9	570	83.7	0.00
	sort_type	2	570	4.24	0.01
	n_inact × sort_type	18	570	0.98	0.48

We also checked the weight-change distributions for the different behavioral tasks because the features of the weight-change distribution may depend on the behavioral task. We analyzed models that leaned toward a working memory task (Romo et al., 1999) in pycog, random dot motion task (Gold and Shadlen, 2007), and multisensory task (Raposo et al., 2014) in pyrl, and delayed non-match to sample task (Simola et al., 2010) in rHebb (Supplementary Figure 3, Supplementary Tables 2, 3). We selected these tasks and set them up based on the original scripts. Most of them showed comparable results, with highly skewed post-mean weight-change distributions and large-positive-kurtosis weight-change distributions. These results suggest that our findings represent the case for a wide range of related cognitive tasks.

We also checked the weight-change distributions for different initial conditions, which may affect the learning-induced weight changes. To enable the comparisons, we set the same initial distribution (Gaussian, mean = 0, standard deviation = 0.15; original settings are described in the Materials and Methods section) and *N*_*rec*_ (= 100) in the HF, pyrl, and rHebb models. Because it was difficult to arrange differences between models, the altered initial distributions were uniform in the pycog model (Supplementary Figure 4; Supplementary Tables 2, 3). Most of them also showed weight-change results comparable to the original settings: the highly skewed post-mean weight-change distributions and large-positive-kurtosis weight-change distributions except the rHebb weight-change distribution did not exhibit significant kurtosis. The weight distribution of the learned HF network maintained a Gaussian distribution, whereas with the default setting it showed high positive kurtosis (Supplementary Figure 1A). When we initialized the synaptic weights of the pyrl model to follow a Gaussian distribution, its distribution remained Gaussian after learning, whereas the post-mean weight-change distribution of the model was highly skewed. The weight distribution of the pycog model was initially uniform; however, after learning, it became highly skewed. These data suggest that all the models showed localized highly plastic synapses in a small population whereas the distribution of weight strength of the learned networks depended on the initial weight distribution.

We finally analyzed the ability of the models to learn with fewer neural units to examine whether smaller networks are sufficient to achieve learning of the context-dependent integration task. In all models, the skewness of the post-mean weight-change distributions and correct choice rates of the task performances decreased as the numbers of neuronal units decreased, although they maintained high correct rates even with small numbers of neuronal units (Supplementary Figure 5, Supplementary Table 4). These results suggest that a large network facilitates learning and that skewness of the post-mean weight-change distributions is associated with task performance.

## Discussion

We analyzed the network structures of four RNN models while performing context-dependent integration tasks. We found that all four models exhibited high positive skewness in post-mean weight-change distributions (Figure 4), and the task performance was sensitive to perturbation in higher plasticity units in most models (Figure 6). No common tendencies among the four models, however, were observed in the final weights after task learning (Figures 2, 3). These results indicate the importance of the plastic changes rather than the constructed connections in performing cognitive tasks.

### Significance of highly plastic changes concentrated in a sparse population

The long-tailed distribution of plastic changes observed in our simulations has been reported in numerous experimental studies. Genes that induce neuronal plasticity (such as c-Fos and Arc) are sparsely expressed in the cerebral cortex and hippocampus. Thus, it was hypothesized that the plastic changes of a small neuronal population mainly represent learning and memory, the so-called engram hypothesis (Hebb, 1949; Tonegawa et al., 2015). In addition, at the synapse level, only a small population shows plastic changes associated with learning (Yang et al., 2009; Hayashi-Takagi et al., 2015). Our findings of high-skewed, post-mean synaptic change distribution and a high-kurtosis synaptic change distribution are comparable to those of previous reports (Figures 4, 5), thus supporting the engram hypothesis (Figure 6).

Moreover, the long-tailed distribution of the task-dependent synaptic change may be explained by the superlinearity of either the activation function or learning rule in each model. In the rHebb model, a superlinear function of the learning rule (but not a linear or sublinear one) leads to sparse and precise synaptic change (Miconi, 2017), which can establish a high-kurtosis synaptic change distribution. Moreover, the pycog and pyrl models use the rectifier activation function (ReLU) to calculate the firing rate. Such a rectifier unit also acts to make neural activity sparse (Glorot et al., 2011), which allows only a few neurons to remain active and plastic.

Our findings regarding the localization of highly plastic synapses in a small population support the hypothesis that RNN systems represent task information in low-dimensional dynamics implemented with their high-dimensional network structures (Barak, 2017). The biologic PFC and the PFC RNN models seemed to pack important information for solving the task in a low-dimensional space (Mante et al., 2013; Sussillo et al., 2015). Packing the information in a few components offers some advantages; e.g., it simplifies the solving strategy (Barak, 2017) and generalizes the task (Neyshabur et al., 2017; Wu et al., 2017). Furthermore, elastic weight consolidation method, preventing catastrophic forgetting, may result in localized changes (Kirkpatrick et al., 2017). It does not, however, suggest that a large network is useless for solving a task. Because the RNN has no prior information of the task, the RNN should represent inputs in high-dimensional space at the beginning of the learning; this offers a computational advantage for dissecting the input patterns (Rigotti et al., 2013). Indeed, most neural network systems are over-parameterized. This redundancy provides benefits in learning speed and memory capacities. We also confirmed that larger network models tend to perform the task better (Supplementary Figure 5B), and that the shift in the performance was accompanied by a shift in the skewness of the post-mean weight-change distribution. Our results thus suggest that the localization of high plastic synapses to a few units is crucial for extracting the low-dimensional essential patterns necessary for the various task representations obtained from high-dimensional spaces.

The exact relationship between the sparseness of weight change and the behavior performance is still unclear. The post-mean weight change in all models are skewed (Figure 4), but only the pycog and HF models, followed by pyrl, showed highly sparse representations, though rHebb did not (Figure 6). There are many candidate factors, which differentiate sparseness of representation among models, such as learning algorithm and plasticity for the external input. Actually, neuronal units showing higher levels of plasticity also tended to exhibit increased plasticity for the external units in the HF, pycog and pyrl models (data not shown), while rHebb cannot change them as a setting. One can imagine that the behavioral importance of the units with more plastic synapses in those three models is, at least in part, due to the input weights onto them being stronger.

In contrast to the above-mentioned properties in synaptic change distributions, the shapes of the synaptic weight distribution after learning were various. It depends on the initial distribution as well as the regularization terms in the objective function. For example, a regularization term can make the distribution sparse and long-tailed from any initial distributions (Lee et al., 2006). The regularization terms of the objective functions, however, are still critical for both learning efficiency (Lee et al., 2006) and task-solving strategies (Sussillo et al., 2015). While data were not shown, models with some initial distribution conditions could not achieve the learning probably because the initial distribution of RNN synaptic weight affects the network dynamics (Sompolinsky et al., 1988). Although our results indicate robustness in the shape of the synaptic change distributions to the initial synaptic distributions (see Supplementary Figure 4), it would be interesting to consider how robust the shape is to alterations in the regularization terms.

### Future study directions

We focused on a context-dependent integration task to determine the necessary structures involved in the process of achieving flexible cognition. Moreover, our findings can be applied to different learning tasks (Supplementary Figure 3). In this study, we limited our analysis to the synaptic weight structures of RNN models. In a subsequent study, we plan to also analyze the dynamics of unit activities during the performance of a task and the underlying learning process. These analyses will provide further insights into how networks encode and establish task information. Furthermore, theoretical investigation will help to elucidate the implications of our findings and establish better RNN models. Recent innovations in RNN optimization methods have enabled computational systems to perform cognitive tasks designed for human and model animals, and have thus allowed for comparisons of the processes occurring in biologic and computational brains (Mante et al., 2013; Cadieu et al., 2014; Yamins et al., 2014; Carnevale et al., 2015; Sussillo et al., 2015). Merging knowledge in both biologic and computational fields that study cognitive tasks will improve our understanding of brain functioning.

## Data availability statement

The datasets analyzed for this study can be found in the Github https://github.com/sakuroki/flexible_RNN.

## Author contributions

SK conceptualized and designed the study. TI modified the study design. SK performed the analyses and programming. SK and TI wrote the draft of the manuscript. All authors read and approved the submitted version.

### Conflict of interest statement

The authors declare that the research was conducted in the absence of any commercial or financial relationships that could be construed as a potential conflict of interest.
