# Supplementary material for: Task-Related Synaptic Changes Localized to Small Neuronal Population in Recurrent Neural Network Cortical Models
Source: Front Comput Neurosci. 2018 Oct 5;12:83. doi: 10.3389/fncom.2018.00083 (PMC6182086; doi:10.3389/fncom.2018.00083)
Supplement: Supplementary file 1 [file Table_1.PDF]

2 Supplementary Tables

Supplementary Table 1. Distribution properties in synaptic weights after the task learning

Model	n	Normality		Skewness			Kurtosis		
		p	W	p	Z	skew.	p	Z	kurt.
HF	10,000	0.00	0.93	0.00	13.9	0.35	0.0	35.9	6.17
pycog (E-E)	14,400	0.00	0.89	0.00	53.8	1.46	0.00	33.7	3.78
pyrl (policy)	10,000	0.00	0.97	0.94	−0.08	0.00	0.00	−34.1	−0.87
rHebb	40,000	0.99	0.99	0.52	−0.64	−0.01	0.94	0.06	0.00
