# Supplementary material for: Task-Related Synaptic Changes Localized to Small Neuronal Population in Recurrent Neural Network Cortical Models
Source: Front Comput Neurosci. 2018 Oct 5;12:83. doi: 10.3389/fncom.2018.00083 (PMC6182086; doi:10.3389/fncom.2018.00083)
Supplement: Supplementary file 2 [file Table_2.PDF]

Supplementary Table 2. Properties in post-mean weight change distributions in different initial states and tasks

Model	n	Normality		Skewness			Kurtosis		
		p	W	p	Z	skew.	p	Z	kurt.
Different task									
pycog (E-E) (working memory)	120	0.00	0.93	0.00	8.17	1.22	0.00	5.70	2.92
pyrl (policy) (random dot)	100	0.01	0.97	0.02	2.37	0.58	0.75	-0.32	-0.26
pyrl (policy) (multisensory)	150	0.04	0.98	0.10	1.66	0.32	0.29	-1.07	-0.41
rHebb (delayed nonmatch)	196	0.01	0.98	0.01	2.70	0.48	0.66	0.44	0.07
Different Distribution									
HF (norm. dist., std = 0.15)	100	0.00	0.91	0.00	4.82	1.41	0.00	3.90	3.86
rHebb (norm. dist., N_{rec}=100)	100	0.00	0.94	0.00	3.24	0.03	0.40	0.84	0.12
pyrl (policy) (norm. dist.)	100	0.00	0.91	0.00	4.67	1.34	0.00	3.77	3.57
pycog (E-E) (Uni. dist.)	120	0.00	0.94	0.00	3.15	0.73	0.48	0.70	0.18
