# Supplementary material for: Task-Related Synaptic Changes Localized to Small Neuronal Population in Recurrent Neural Network Cortical Models
Source: Front Comput Neurosci. 2018 Oct 5;12:83. doi: 10.3389/fncom.2018.00083 (PMC6182086; doi:10.3389/fncom.2018.00083)
Supplement: Supplementary file 3 [file Table_3.PDF]

Supplementary Table 3. Distribution properties in weight changes in different initial states and tasks

Model	n	Normality		Skewness			Kurtosis		
		p	W	p	Z	skew.	p	Z	kurt.
Different task									
pycog (E-E)<br>(working memory)	15,881	0.00	0.82	0.00	84.4	2.99	0.00	74.1	58.6
pyrl (policy)<br>(random dot)	1,000	0.00	0.99	0.60	-0.53	-0.04	0.00	5.85	1.45
pyrl (policy)<br>(multisensory)	2,250	0.00	0.99	0.00	2.96	0.15	0.00	8.93	1.55
rHebb<br>(delayed nonmatch)	38,461	0.00	0.99	0.48	0.71	0.01	0.00	8.90	0.25
Different Distribution									
HF<br>(norm. dist., std = 0.15)	10,000	0.00	0.94	0.19	1.32	0.03	0.00	33.9	5.27
rHebb<br>(norm. dist., N_{rec}=100)	9,216	0.18	0.99	0.18	1.33	0.33	0.52	0.65	0.03
pyrl (policy)<br>(norm. dist.)	10,000	0.00	0.98	0.00	14.6	0.37	0.00	23.0	2.24
pycog (E-E)<br>(Uni. dist.)	14,400	0.00	0.67	0.00	96.0	4.44	0.00	65.7	35.3
