# Supplementary material for: Task-Related Synaptic Changes Localized to Small Neuronal Population in Recurrent Neural Network Cortical Models
Source: Front Comput Neurosci. 2018 Oct 5;12:83. doi: 10.3389/fncom.2018.00083 (PMC6182086; doi:10.3389/fncom.2018.00083)
Supplement: Supplementary file 4 [file Table_4.PDF]

Supplementary Table 4. Kruskal-Wallis test results of the smaller network learning experiments

Parameter	Model	n	df	H	p
Skewness	HF	5	4	9.66	0.05
	pycog	5	5	12.3	0.03
	pyrl	5	2	1.85	0.40
	rHebb	5	5	19.6	0.00
Correct rate	HF	5	6	21.4	0.00
	pycog	5	5	15.8	0.01
	pyrl	5	2	6.02	0.05
	rHebb	5	5	19.8	0.00
