# Supplementary material for: Task-Related Synaptic Changes Localized to Small Neuronal Population in Recurrent Neural Network Cortical Models
Source: Front Comput Neurosci. 2018 Oct 5;12:83. doi: 10.3389/fncom.2018.00083 (PMC6182086; doi:10.3389/fncom.2018.00083)
Supplement: Supplementary file 5 [file Image_1.PDF]

1 Supplementary Figures

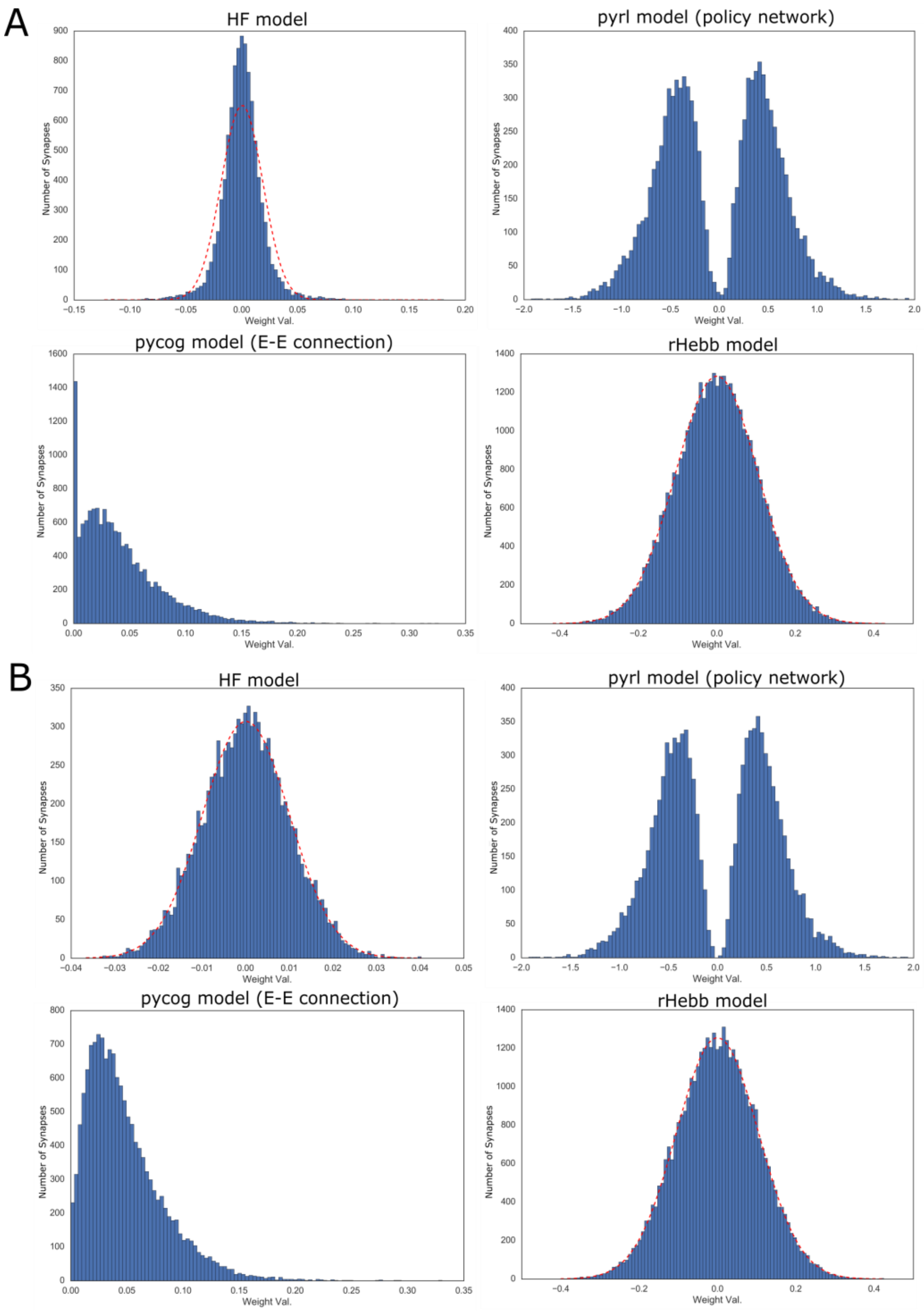

Supplementary Figure 1. Distribution of synaptic weights after (A) and before (B) the task learning. The dotted red line in the HF and rHebb models represents a normal distribution with mean and sigma of the weight values.
