# Supplementary material for: Task-Related Synaptic Changes Localized to Small Neuronal Population in Recurrent Neural Network Cortical Models
Source: Front Comput Neurosci. 2018 Oct 5;12:83. doi: 10.3389/fncom.2018.00083 (PMC6182086; doi:10.3389/fncom.2018.00083)
Supplement: Supplementary file 6 [file Image_2.PDF]

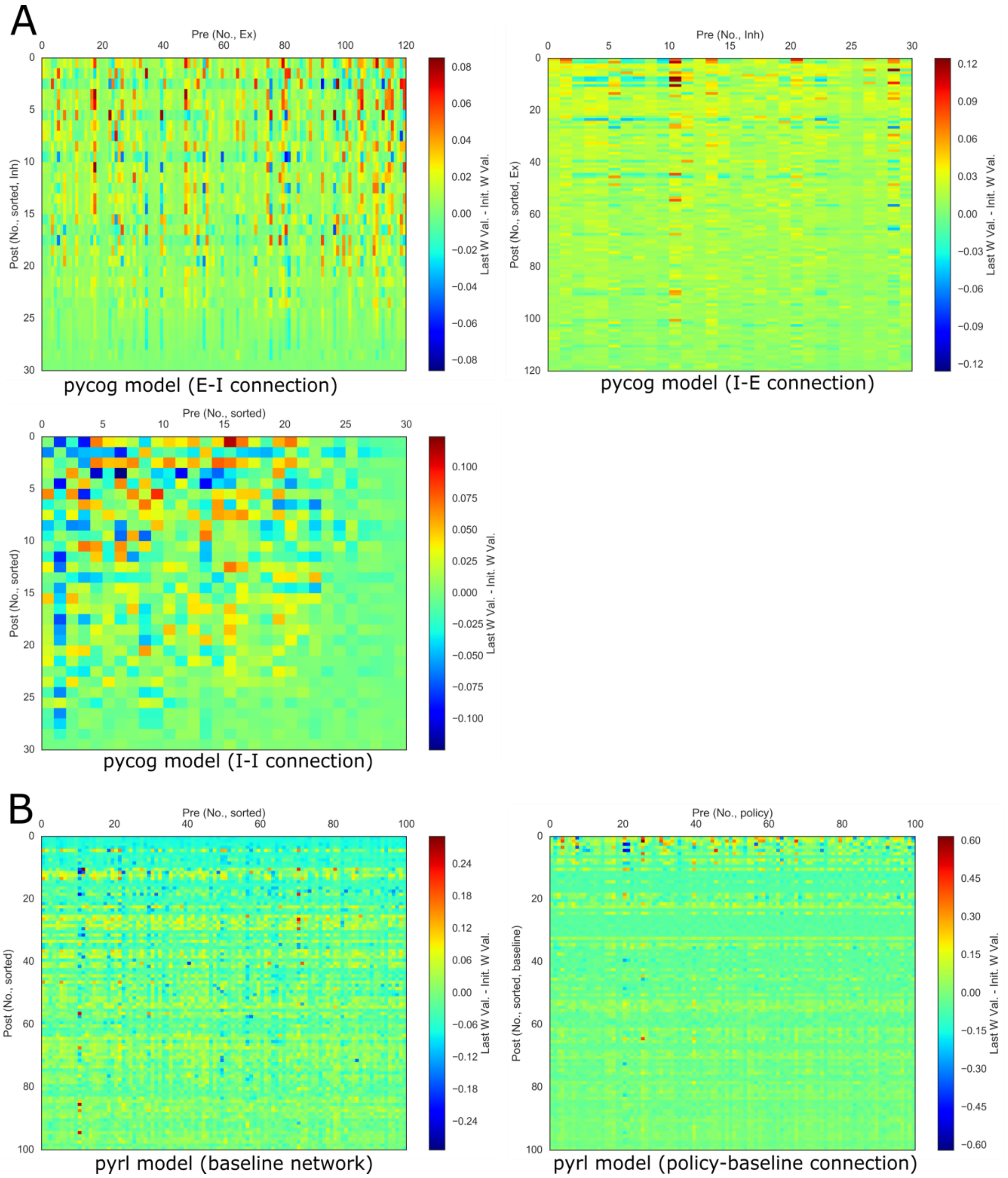

Supplementary Figure 2. Weight change plots of (A) pycog I-I, I-E, E-I, and (B) pyrl baseline-baseline and policy-baseline connections, sorted by post-mean weight changes.
