# Supplementary material for: Task-Related Synaptic Changes Localized to Small Neuronal Population in Recurrent Neural Network Cortical Models
Source: Front Comput Neurosci. 2018 Oct 5;12:83. doi: 10.3389/fncom.2018.00083 (PMC6182086; doi:10.3389/fncom.2018.00083)
Supplement: Supplementary file 7 [file Image_3.PDF]

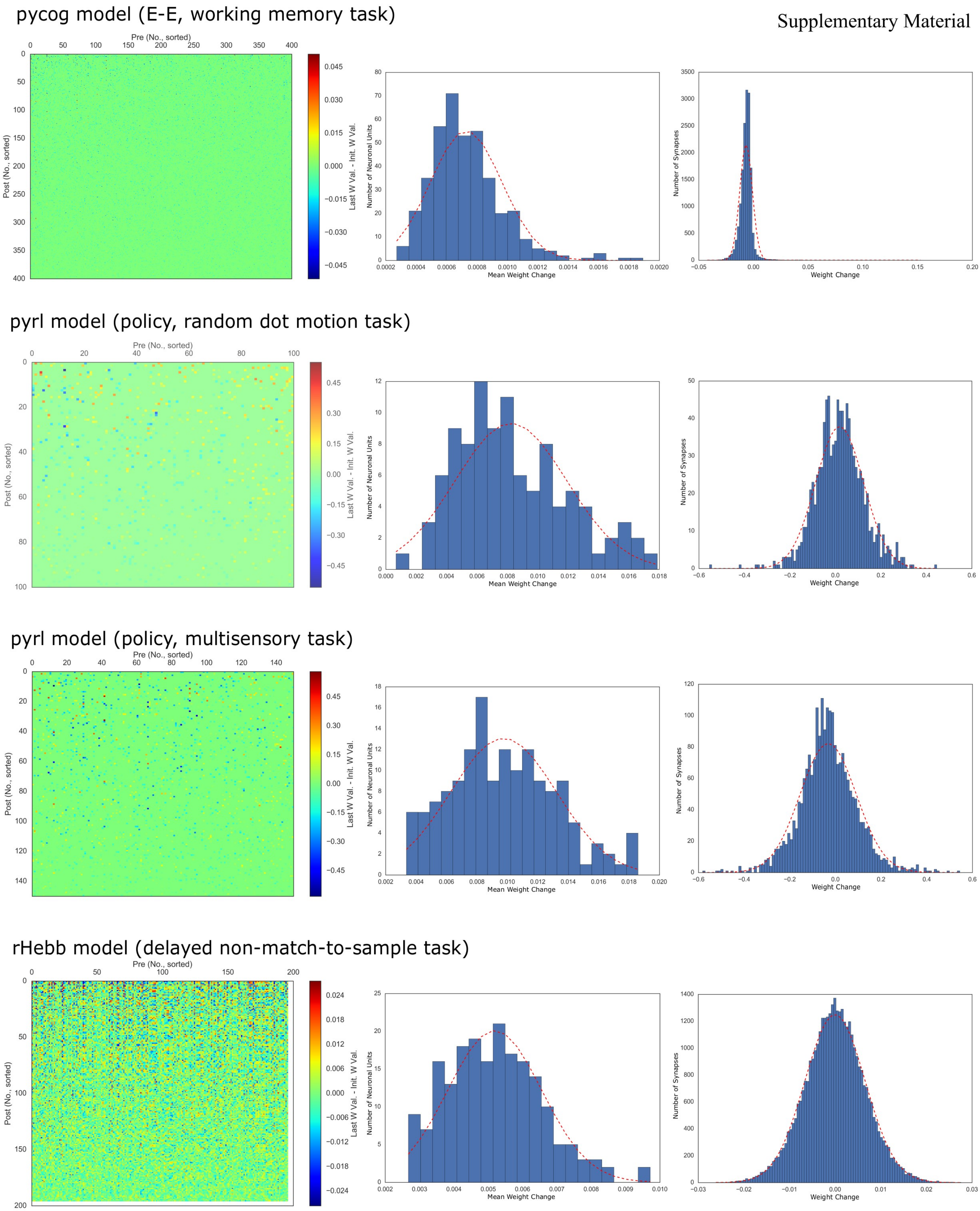

Supplementary Figure 3. Sorted weight changes plot (left), post-mean weight change (middle) and weight change (right) distributions performing different cognitive tasks. References for each task are shown below (working memory (Romo, Brody, Hernández, & Lemus, 1999), random dot motion (Gold and Shadlen, 2007), multisensory (Raposo et al., 2014), delayed nonmatch to sample (Simola et al., 2010)).
