# Supplementary material for: Task-Related Synaptic Changes Localized to Small Neuronal Population in Recurrent Neural Network Cortical Models
Source: Front Comput Neurosci. 2018 Oct 5;12:83. doi: 10.3389/fncom.2018.00083 (PMC6182086; doi:10.3389/fncom.2018.00083)
Supplement: Supplementary file 8 [file Image_4.PDF]

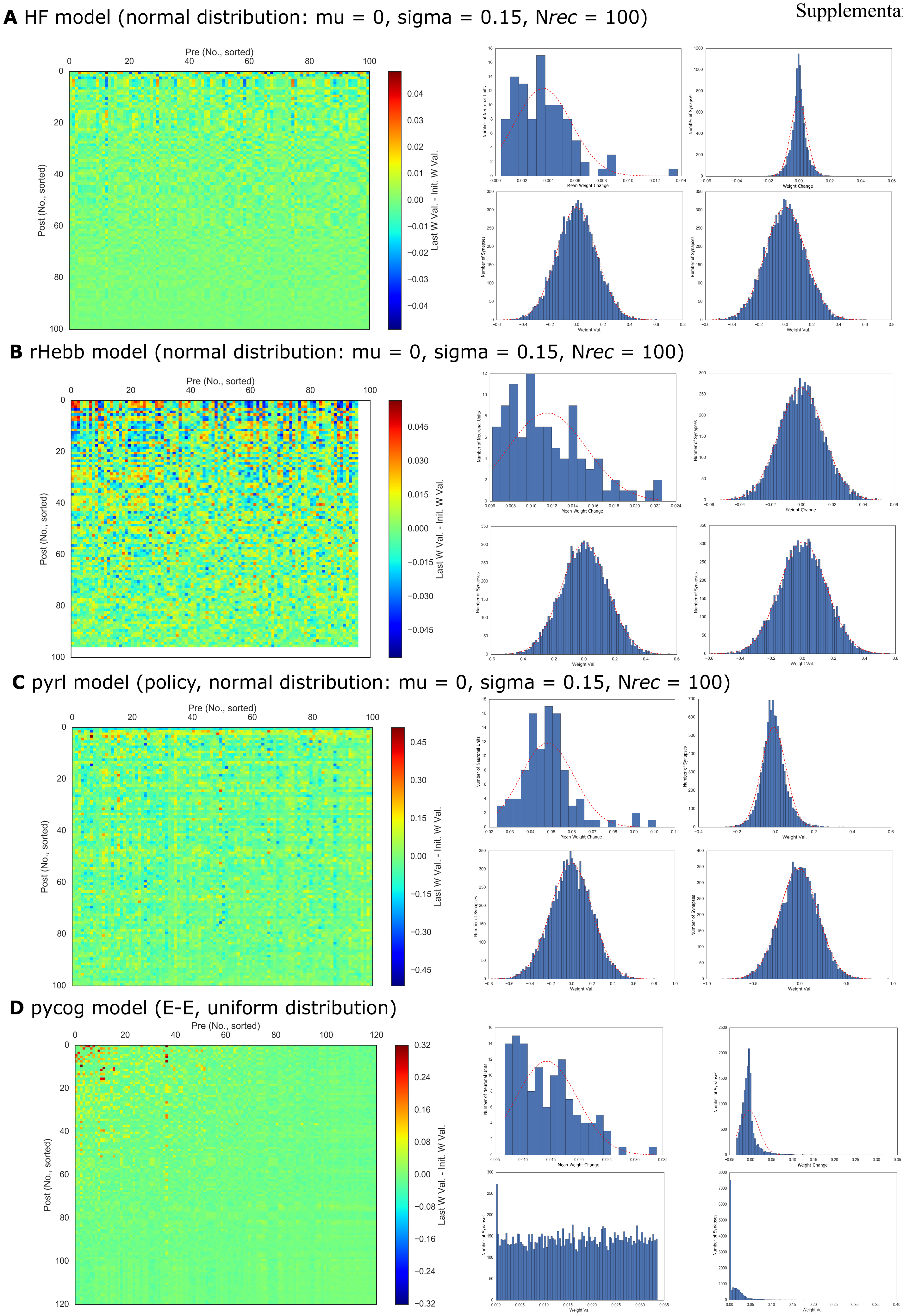

Supplementary Figure 4. Sorted weight change plot (left), post-mean weight change (upper middle), weight change (upper right) distributions with different initial weight distributions. Lower left and right panels show the initial and last weight value distributions, respectively.
