# Supplementary material for: Task-Related Synaptic Changes Localized to Small Neuronal Population in Recurrent Neural Network Cortical Models
Source: Front Comput Neurosci. 2018 Oct 5;12:83. doi: 10.3389/fncom.2018.00083 (PMC6182086; doi:10.3389/fncom.2018.00083)
Supplement: Supplementary file 9 [file Image_5.PDF]

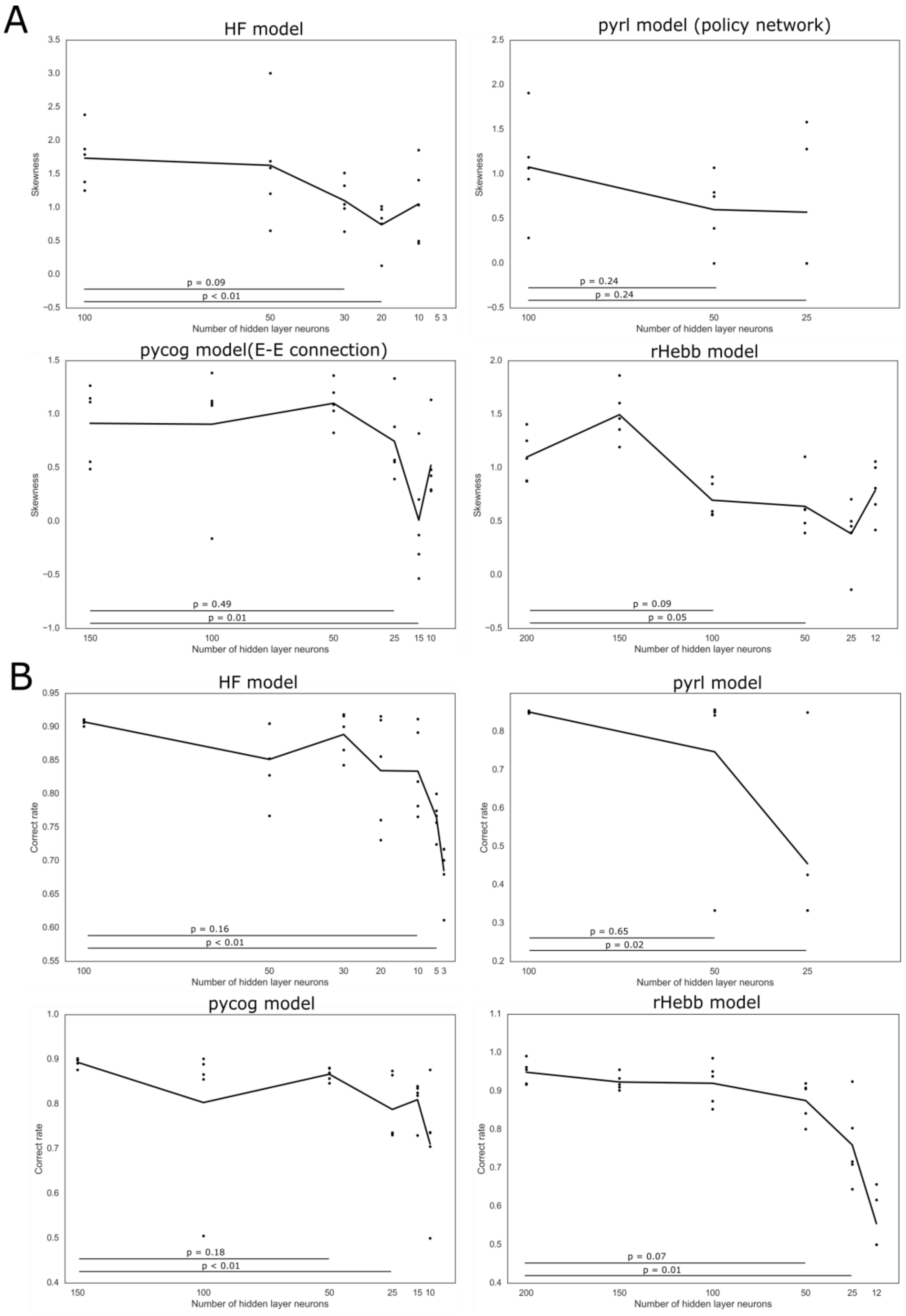

Supplementary Figure 5. Skewness of post-mean weight change distribution (A) and correct rates (B) of each model with fewer neuronal units. Dots indicates values of individual systems and line indicates the means.
